# Circadian Pacemaking in Cells and Circuits of the Suprachiasmatic Nucleus

**DOI:** 10.1111/jne.12125

**Published:** 2014-01-13

**Authors:** M H Hastings, M Brancaccio, E S Maywood

**Affiliations:** Division of Neurobiology, MRC Laboratory of Molecular BiologyCambridge, UK

**Keywords:** VIP, DREADD, pharmacogenetic, paracrine, sleep

## Abstract

The suprachiasmatic nucleus (SCN) of the hypothalamus is the principal circadian pacemaker of the brain. It co-ordinates the daily rhythms of sleep and wakefulness, as well as physiology and behaviour, that set the tempo to our lives. Disturbance of this daily pattern, most acutely with jet-lag but more insidiously with rotational shift-work, can have severely deleterious effects for mental function and long-term health. The present review considers recent developments in our understanding of the properties of the SCN that make it a robust circadian time-keeper. It first focuses on the intracellular transcriptional/ translational feedback loops (TTFL) that constitute the cellular clockwork of the SCN neurone. Daily timing by these loops pivots around the negative regulation of the *Period* (*Per*) and *Cryptochrome* (*Cry*) genes by their protein products. The period of the circadian cycle is set by the relative stability of Per and Cry proteins, and this can be controlled by both genetic and pharmacological interventions. It then considers the function of these feedback loops in the context of cytosolic signalling by cAMP and intracellular calcium ([Ca^2+^]_i_), which are both outputs from, and inputs to, the TTFL, as well as the critical role of vasoactive intestinal peptide (VIP) signalling in synchronising cellular clocks across the SCN. Synchronisation by VIP in the SCN is paracrine, operating over an unconventionally long time frame (i.e. 24 h) and wide spatial domain, mediated via the cytosolic pathways upstream of the TTFL. Finally, we show how intersectional pharmacogenetics can be used to control G-protein-coupled signalling in individual SCN neurones, and how manipulation of Gq/[Ca^2+^]_i_-signalling in VIP neurones can re-programme the circuit-level encoding of circadian time. Circadian pacemaking in the SCN therefore provides an unrivalled context in which to understand how a complex, adaptive behaviour can be organised by the dynamic activity of a relatively few gene products, operating in a clearly defined neuronal circuit, with both cell-autonomous and emergent, circuit-level properties.

Circadian rhythms are those daily cycles of behaviour and physiology that persist when an individual human subject, an experimental animal or a plant, is isolated in a time-free environment. Their persistence betrays the presence of an internal clock that is able, autonomously, to define periods of approximately (*circa-*) 1 day (*-dian*). Under natural conditions, these circadian clocks are entrained to the cycle of light and darkness, so that they enable the physiology and behaviour of the organism to anticipate, and thereby adapt to, the solar day and night. In mammals, the principal pacemaker in the brain is the suprachiasmatic nucleus (SCN) 1 and this receives direct innervation from specialised ganglion cells of the retina that mediate entrainment of the clock by light 2. The role of the SCN is to generate a stable internal representation of solar time, and then to convey that via neural, behavioural and endocrine pathways to co-ordinate all aspects of daily functions across the brain and body. Importantly, the SCN can generate daily time autonomously; the retinal innervation serves solely to synchronise the circadian oscillator, not to sustain it. In this regard, the SCN is a remarkable piece of neurobiology: an unparalleled exemplar of localisation of autonomous function in the central nervous system.

With the discovery of the molecular feedback loops that constitute the core circadian time-keeper (see below), recognition of the medical relevance of circadian clocks has burgeoned. First, all major organs have local circadian clock mechanisms; the SCN is not the sole clock, although it is the orchestrator of innumerable clocks distributed across the body 3. Second, these tissue-based clocks drive the circadian expression of approximately 10% of the genes and proteins expressed locally in a particular tissue 4,5. Consequently, vital metabolic processes such as hepatic nitrogen metabolism, gluconeogenesis, cardiovascular function and renal de-toxification all follow precisely defined, interlocking cycles that optimise metabolic performance. The capabilities of brain and body therefore vary as a function of circadian time; thus, we should look upon ourselves as 24-h machines. The value of this circadian machinery is readily taken for granted and ignored but becomes most evident when it is disrupted, with the archetypal example being ‘jet-lag’ following time-zone transitions. The consequential misalignment of local clocks with each other and with environmental time, as well as the temporal scramble that occurs during their progressive re-adjustment, is reflected in the various aspects of tiredness, mental confusion and general dysphoria. A far more insidious threat to public health arising from clock disruption comes from rotational shift-work, with epidemiological studies revealing significantly increased risks of cancer, cardiovascular disease and obesity, as well as diabetes 6. Furthermore, animal-based studies have revealed the mechanistic links behind these phenomena, with, for example, circadian disruption leading to insulin resistance 7,8. Put simply, if the liver, pancreas and skeletal muscle are not working in time and in tune, effective regulation of blood glucose and insulin is compromised. In addition, there has been a longstanding recognition that psychiatric conditions, especially major depressive disorders, are affected by (and in turn affect) circadian processes 9. Although mechanistic links remain elusive, animal models have again provided novel insights into how clocks, light, sleep and mood may interact 10. More immediate in terms of public health is the impact of the loss of tight circadian control of sleep on the care and life quality of patients with neurodegenerative diseases 11. It is the difficulties involved in trying to care for someone without a regular sleep cycle in a home setting that is the principal cause of institutionalisation, with its incumbent personal, social and economic costs. The hope, therefore, is that by determining the basic molecular and neurobiological mechanisms that govern circadian pacemaking, not only will an engaging piece of biology be decoded, but also new opportunities will be presented to address diseases characteristic of modern society.

## A molecular pacemaker built around feedback loops

**Fig. 1:**
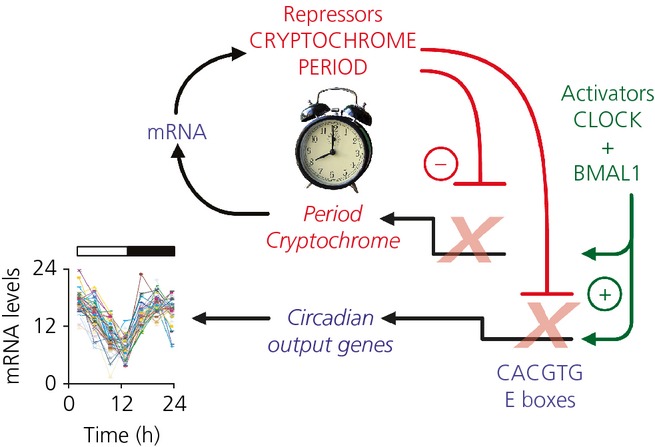
A schematic view of the core molecular feedback loop that sits at the heart of the mammalian pacemaker. The definition of circadian time pivots around the activation of *Per* and *Cry* genes by Clock/Bmal1 heterodimers (acting at E-box enhancer sequences), alternating with repression of the same genes by their protein products. Clock-controlled output genes carrying E-boxes are also subject to daily activation and repression, generating downstream transcriptional cascades that ultimately encode circadian cycles of physiology and behaviour.

At a molecular level, the core oscillatory mechanism of the SCN commences with trans-activation of *Per* and *Cry* genes by heterodimers of Clock and Bmal1, basic helix-loop-helix transcription factors that associate via so-called PAS dimerisation domains, and act via E-box enhancer elements in their target genes 12,13 (Fig. 1). Over the course of the circadian morning, the levels of *Per* and *Cry* mRNA accumulate in SCN neurones and, by the end of the circadian day, Per and Cry proteins appear, form complexes and start to enter the nucleus where they interfere with the actions of Clock and Bmal1, in part by recruiting transcriptional inhibitory complexes. As circadian night progresses, mRNA levels fall, translation of Per and Cry proteins declines, and existing Per/Cry complexes are actively degraded: a process essential for clock progression. This ultimately releases E-boxes from negative regulation and the cycle is ready to start anew with a new circadian day. This central feedback oscillation is augmented by additional loops involving the *Rev-Erba*, *Rev-Erbb* and *RORA* genes, which are also driven by Clock/Bmal1, and their protein products in turn drive rhythmic expression of *Bmal1* via RORE sequences 14. The combined loss of these genes renders mice behaviourally arrhythmic, as does the loss of *Cry1* and *Cry2*, *Bmal1* alone, or *Per1* and *Per2* in combination 1,15.

A further category of clock-regulated genes, the PAR bZIP transcription factors that include Dbp, contributes less to the core oscillator and more to the timing the expression of clock-controlled genes that carry their D-box regulatory elements 16. These output genes are downstream of the oscillator and are ultimately responsible for generating the circadian cycles of cellular activity that underpin circadian behaviour and physiology. In the SCN, this output includes genes and proteins involved in synaptic transmission, metabolism and electrophysiological activity (ion channel and receptors) as the neurones alternate between nocturnal quiescence (firing < 1 Hz) and high spontaneous activity during circadian daytime (up to 10 Hz) 17,18. Through these changes in firing, neuropeptidergic and GABAergic drive to SCN target neurones will co-ordinate the autonomic and neuroendocrine cascades that ultimately synchronise the activity of local clocks distributed in peripheral tissues and other brain regions. These clocks in turn, via the same core mechanisms, will control local transcriptional cascades to direct organ-specific physiology 19. For example, circadian cues in the liver drive the rhythmic expression of the transcription factor HNF4a, a vital regulator of liver-specific genes involved in nitrogen metabolism 20. In SCN-ablated mice, circadian regulation of *HNF4a* and its downstream transcriptional cascade is lost.

The discovery of circadian clock genes and their output cascades has propelled the development of new technologies for the analysis of clock function. In particular, real-time imaging of circadian transcription and protein expression using bioluminescent and fluorescent reporters has brought entirely novel views of circadian organisation (see Supporting information, Video S1). For example, SCN slices from mice carrying *Per1-luciferase* 21, *Cry1-luciferase* 22 or *Per1-EGFP* transcriptional reporter transgenes 23 or the Per2::LUC fusion protein reporter 3 have been used to explore the cell-autonomous and circuit-level properties of the transcriptional/ translational feedback loops (TTFL). In particular, this has shown that circadian gene expression progresses as a spatio-temporal wave across the SCN: the TTFLs of the individually rhythmic cells are synchronised across the circuit, although they do not peak simultaneously. Rather they hold stereotypical phase relationships with each other, the more dorsal SCN cells being phase-advanced by 2–3 h 24 relative to the rest. These waves are dependent on the ability of SCN neurones to communicate via tetrodotoxin (TTX)-sensitive action potentials 21 and are also disrupted *ex vivo* after exposure *in vivo* to constant light, a treatment that, commonly, renders mice behaviourally arrhythmic 23, likely because of continuous activation of electrical firing and *Per* gene expression. The second level of organisation revealed by bioluminescent [*Per1-luc* transcriptional 25 and Per2::LUC fusion protein 3] reporters has been the activity of local clocks. First, this showed that all major organs, when isolated in a dish, could nevertheless express very clear circadian cycles, demonstrating that conserved clock functions are distributed across the entire organism. Second, they have helped to define the mechanisms (e.g. corticosteroid rhythms, temperature rhythms, periodic feeding) that act *in vivo* and *in vitro* to entrain these local clocks and thereby establish circadian coherence across the animal. Finally, by testing the ability of different cis-regulatory elements to drive luciferase expression in cell culture, it has been possible to assemble a molecular phase map of progressive and inter-linked gene expression patterns around the circadian cycle. In particular, circadian phase progression is characterised by sequential activation of E-boxes, D-boxes and RORE sequences 26. Circadian timing is therefore a pervasive feature of biological integration that extends across space (anatomy) and time.

## Setting the period of the TTFL pacemaker: protein stability and drugs

An approximately 24-h intrinsic period is a canonical feature of a circadian pacemaker. Identification of the core mechanism as a negative-feedback loop has made it possible to identify points in the cycle that contribute to setting period length. Indeed, the definitive mouse mutant *Clock* was revealed as a long period phenotype that was subsequently shown to arise from impaired transactivation by the mutant Clock protein lacking exon 19 27. The reduced transcription rate of *Per* and *Cry* target genes in the SCN thus leads to a slower progression of the cycle and longer behavioural (and other) rhythms. More recently, a variety of small molecule screens and RNA interference studies in fibroblasts have identified factors involved in both transcriptional activation and repression as control points, as well enzymes that affect Per and Cry stability 28–30. Similar conclusions have arisen from the study of spontaneous and ENU-induced mutations in hamsters and mice, as well as in humans with specific sleep phase disorders. For example, loss of function of the E3 ubiquitin-ligases Fbxl3 and Fbxl21 can delay or accelerate proteasomal degradation of Cry1 and Cry2, leading to correspondingly longer or shorter circadian periods, monitored in mice *in vivo* or in tissue and cell culture 31,32. Another point of control of clock speed comes from the role of casein kinase 1 enzymes (CK1*ε*/*δ*) in phosphorylating Per proteins and thereby licensing them for proteasomal degradation. Mutations in *mCK1ε*, *hCK1δ* or in *hPer2* that accelerate degradation concomitantly shorten the circadian period 33. For example *CK1ε*^*Tau*^ mutant mice exhibit 20-h activity rest cycles and bioluminescence rhythms in SCN and peripheral tissues because more rapid degradation of Per proteins terminates the negative-feedback phase earlier than in wild-types 34. These findings present new possibilities for therapeutic regulation and, indeed, proof of principle has been achieved with inhibitors of CK1*ε* and CK1*δ*. Not only do these compounds dose-dependently slow down Per degradation and lengthen circadian period in wild-type mice and tissue cultures, but also they can reverse the period shortening effects of the *CK1ε*^*Tau*^ mutation both in SCN slices in culture and in the mouse *in vivo* 35. Thus, by titrating gene dose and the degree of pharmacological inhibition of CK1*ε*/*δ*, it is possible to generate SCN with periods ranging from 20 to 30 h. Over this entire range, the SCN molecular pacemaker nevertheless remains extremely precise and runs with high amplitude: indicative of its remarkable robustness. It is only with the highest doses of CK1*ε*/*δ* inhibition that Per cannot be cleared effectively and the oscillation grinds to a halt. A comparable range of stable circadian periods can also be achieved by inter-crossing mice carrying the *CK1ε*^*Tau*^ and *Fbxl3*^*Afh*^ mutations. The latter slows down Cry degradation and thereby lengthens circadian period, although this effect is not dominant over the shortening of period by *Tau*. Rather, assortment of *Tau* and *Afh* alleles leads to additive and independent tuning of the period of circadian behaviour and SCN gene expression, extending between 20 and 28 h, and with a range of different genotypes capable of generating a conventional wild-type period of approximately 24 h 36. Finally, the *Afh* mutation alone can give a range of effects, depending upon the availability of its Cry substrates, insofar as in a Cry1-null background, its period lengthening mediated via Cry2 is several hours shorter than that seen in Cry2-null mice where the more potent Cry1 is the mediator of period lengthening. Again, by assorting *Afh*, *Cry1*^−/−^ and *Cry2*^−/−^ alleles, stable behavioural and SCN periods ranging between 18 and 29 h can be generated 37. It is likely that the range of circadian periods and chronotypes observed in human populations 38, with the exception of rare single-allele defects such as familial advanced sleep phase syndrome 39, arises from such multigenic interactions.

## Expanding the TTFL pacemaker: a day in the life of an SCN neurone

**Fig. 2:**
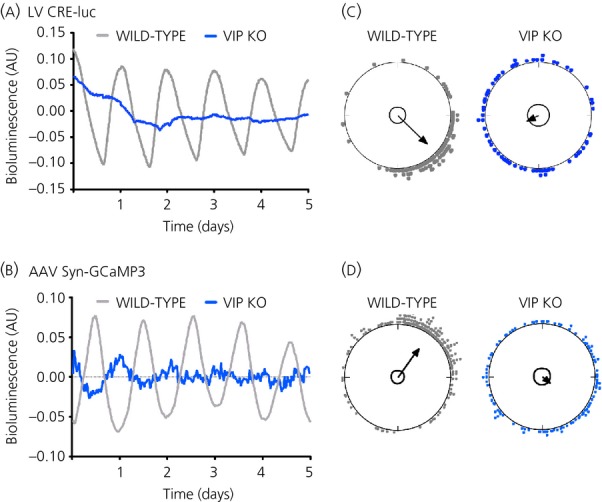
Circadian rhythms of cellular [Ca^2+^]_i_ and CRE-dependent transcription in the suprachiasmatic nucleus (SCN), reported by adeno-associated virus GCamp3 and LV CRE-luciferase, respectively. Note that both types of circadian rhythm are lost in SCN lacking the neuropeptide vasoactive intestinal peptide (VIP) (a, b) and this arises from loss of cellular synchrony across the slice, as revealed by Rayleigh plots of the phases of individual neurones (c, d). Redrawn with permission from Brancaccio *et al*. 45 AU, arbitary units; KO, knockout.

**Fig. 3:**
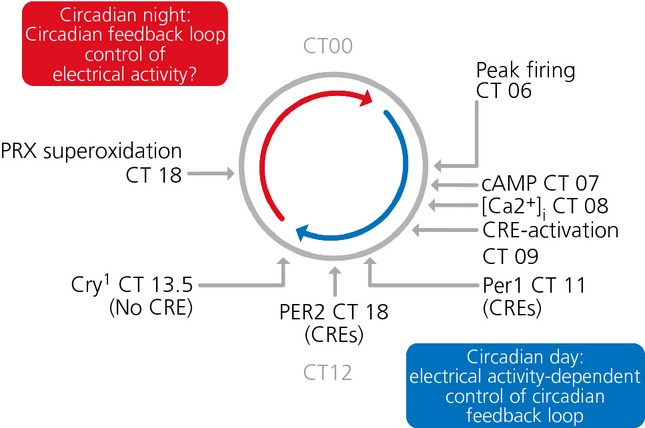
A schematic representation of the phase map of cytosolic and transcriptional events within the suprachiasmatic nucleus clockwork. Electrophysiological activity peaks in the circadian day, followed by peak cAMP, [Ca^2+^]_i_ and CRE-dependent transcription. The expression of *Per* genes, which carry CREs then follows, whereas *Cry1* expression, mediated by via E-boxes but not CREs, lags behind. Circadian night is characterised by electrical quiescence and altered metabolic state, as revealed by the peak of peroxiredoxin (PRX) superoxidation. Redrawn with permission from Brancaccio *et al*. 45 and Edgar *et al*. 52.

The TTFL model as described above is self-contained and the circadian time signal that it generates is sent out via clock-controlled genes. This, however, says nothing about the inputs to the loop. As noted above, treatment with TTX to compromise the electrophysiological activity of the SCN rapidly supresses the amplitude of the TTFL, as reflected by bioluminescent and fluorescent *Per* gene expression, which is seen to fall dramatically 21,40. Thus, there must be a tight linkage between events at the neuronal membrane and components of the TTFL. One example of this comes from the resetting effect mediated by retinal illumination. Acting via NMDA and non-NMDA receptors, glutamate, released by the ganglion cell terminals in the core region of the SCN, stimulates firing of action potentials, increases intracellular calcium levels [Ca^2+^]_i_, activates the calcium/cAMP-dependent transcription factor CREB and increases *Per* gene expression via calcium/cAMP-dependent response elements (CRE) 41,42. The effect of the resulting bolus of Per protein is to reset the ongoing oscillation of the TTFL, causing it to delay or advance if light is encountered in early or late circadian night, respectively 43. This specific example of resetting appears to highlight a more general relationship between the TTFL and electrical activity within the free-running oscillator. The spontaneous firing rate of neurones in organotypic SCN slices peaks around the middle of circadian day (CT06). This accompanies or slightly precedes peaks in the levels of cAMP, as measured by immunoassay 24,44, and [Ca^2+^]_i_ reported by a genetically encoded reporter GCaMP3, delivered to the slices by adeno-associated virus (AAV) 45 (Fig. 2a; see also Supporting information, Video S1). If electrical activity is compromised with TTX, the circadian surge in [Ca^2+^]_i_ is lost. In untreated SCN, the peaks of cAMP and [Ca^2+^]_i_ are followed by a circadian peak in CRE-mediated transcription, as reported by a lenti-virus CRE-luciferase reporter 45 (Fig. 2b). Again, TTX flattens the circadian rhythm of CRE activation, revealing its dependence on the electrical activity of the SCN neurone. The significance of the CRE activity is that the bioluminescent reporters for *Per1* and *Per2* carrying CREs are expressed soon after the CRE activity peak, whereas a *Cry1* sequence that does not carry CREs peaks some hours later 22 (Fig. 3). Thus, although the E-boxes present in *Per* and *Cry* may be important for generic circadian expression, the temporal sequence of genes carrying E-boxes, as well as the responsiveness of the TTFL to resetting cues, may be determined by the presence or absence of CRE sequences, which link firing rate and [Ca^2+^]_i_ with the induction of *Per* gene expression.

If circadian day is defined by electrical activity, peak [Ca^2+^]_i_/CRE signalling and circadian gene expression, what typifies circadian night in the SCN neurone? A fall in resting membrane potential will impose electrical quiescence and this may in part be mediated by up-regulation of large conductance, Ca^2+^- and voltage-activated (BK) potassium channels 46. How this up-regulation is achieved is not clear, although temporal mis-expression of BK channels with a transgene carrying a day-specific *Per1* promoter has shown that day-time down-regulation of the BK current is required for the high amplitude rhythm of electrical activity in the SCN, as well as for the restriction of locomotor behaviour to an appropriate nocturnal phase 47. A further consideration is the metabolic state of the SCN. In other tissues, it is clear that circadian changes in redox homeostasis and NAD(P)^+^ production can feedback into the core clock mechanism, acting via NAD^+^-dependent deacetylase Sirt1 to tune the expression of metabolic genes 48,49. In this way, the clock mechanism can buffer the cell against daily swings in nutrient supply. Thus, in contrast to the daytime surge in firing, the nocturnal quiescence of SCN neurones may be a phase of lower metabolic demand, as demonstrated by reduced uptake of 2-deoxyglucose 50. This lower metabolic state may re-programme the dynamics of the TTF as it progresses through its interval of negative-feedback. Even in the absence of a TTFL, mammalian erythrocytes can express circadian cycles of superoxidation of peroxiredoxin (Prx) protein, part of the cellular defence against reactive oxygen species 51. Indeed, it has been shown that such metabolic rhythms are conserved across all forms of life: eukaryotic and prokaryotic, and may pre-date TTFL mechanisms as a ‘proto-clock’ 52. Organotypic slices of the SCN also express a high amplitude cycle of Prx superoxidation that peaks during circadian night 52. It remains to be determined whether this solely represents a metabolic output of the TTFL, whether it influences the TTFL, or whether it plays a parallel/independent role in SCN circadian pacemaking.

## Holding the SCN clock cells together: synchronisation by paracrine neuropeptidergic signals

**Fig. 4:**
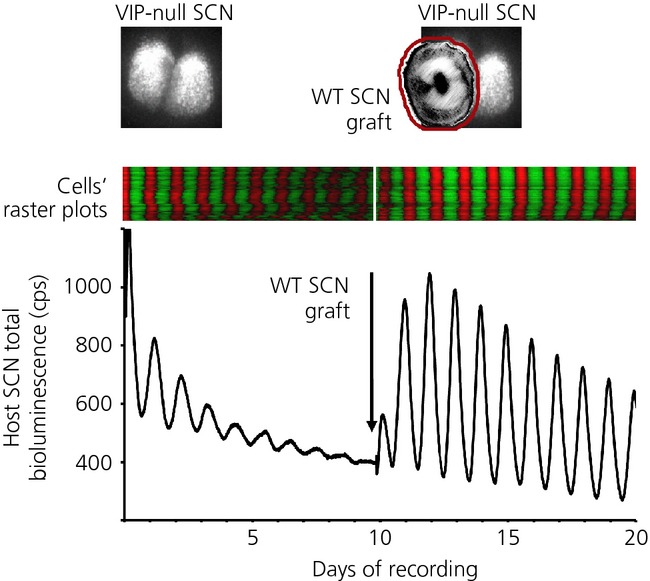
Co-culture reveals the role of paracrine vasoactive intestinal peptide (VIP) signalling in maintaining coherent circadian gene expression in the suprachiasmatic nucleus (SCN). Upper panels depict VIP-null, Per2::luc SCN (left) and then addition of nonbioluminescent wild-type (WT) graft onto mutant host (right). Middle panels present raster plots of the bioluminescent gene expression in host SCN, before (left) and after grafting (right). The lower trace indicates the aggregate bioluminescent signal recorded from VIP-null host SCN before (left) and after (right) the addition of the graft. Note rapid re-organisation of circadian gene expression following grafting. Redrawn with permission from Maywood *et al*. 56.

The spatio-temporal waves of circadian gene expression observed in SCN organotypic slices reveal a sophisticated and complex form of synchronisation of the TTFLs resident within the many individual ‘clock’ neurones. This synchrony is disrupted by interference with synaptic communication across the slice; for example, by blocking action potentials with TTX 21,40 or by compromising synaptic vesicle cycling with botulinum toxin 17, although the question remains regarding what endogenous neurotransmitters are involved? The retinorecipient neurones of the SCN core region are characterised by the neuropeptide vasoactive intestinal peptide (VIP), and mice lacking the VPAC2 receptor for VIP have a grossly disordered circadian behaviour and physiology 53. Moreover, the circadian bioluminescence rhythms of SCN are of low amplitude and are not synchronised across the circuit (Fig. 4) 54. Gene expression can be activated and synchrony transiently imposed, however, by treatment with a second SCN neuropeptide, gastrin-releasing peptide (GRP), or forskolin, an activator of adenylyl cyclase and thus cAMP production. In VIP-null SCN, the desynchronised phenotype can be transiently reversed by a bolus application of VIP 55.

To explore further the mechanism of action of VIP and other neuropeptides in mediating interneuronal synchrony, we developed a novel SCN graft protocol in which circadian gene expression was first recorded from a mutant SCN carrying a genetically encoded bioluminescence reporter, and then a second wild-type ‘graft’ SCN lacking a reporter was placed on top of the ‘host’ and the recording continued 56. When the host was a VIP-null SCN, the effects were immediate and obvious; within 24 h, the amplitude of cellular circadian gene expression in the host neurones was elevated and synchrony restored (Fig. 4). The effect was stable, with some recordings lasting well beyond 10 days. Critically, the effect was truly paracrine because the presence of a 10-kDa molecular weight cut-off membrane between host and graft that would preclude neuronal contact did not interfere with it. By contrast, a 2-kDa cut-off membrane that would prevent the diffusion of VIP from graft to host did prevent synchronisation of the host VIP-null SCN, although synchronisation did start following inversion of the graft/membrane to allow the graft direct contact with the host. Furthermore, the synchronising effect of the paracrine signals represented a true signalling of time from graft to host: it was not simply a permissive condition for the TTFL of the host to express its intrinsic period. When *Tau* mutant grafts were applied, they established a 20-h period rhythm in the host, whereas *Afh* mutant grafts drove the host at 28 h 56.

**Fig. 5:**
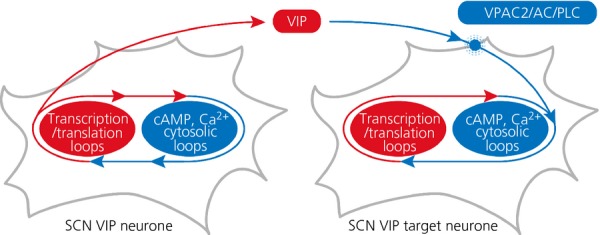
Schematic model to illustrate how vasoactive intestinal peptide (VIP) acting via VPAC2 receptors is the binding factor between suprachiasmatic nucleus neurones, linking the transcriptional/ translational feedback loops (TTFL) on one cell to cytosolic signalling pathways upstream of the TTFL in target neurones.

Thus, the co-culture paradigm revealed VIP-mediated paracrine signalling of circadian time, with VIP neurones capable of imposing a heterotypical period on an ectopic SCN circuit. In addition, it also revealed a VIP-independent pathway, insofar as when the host SCN lacked VPAC2 receptor and was therefore ‘blind’ to graft-derived VIP, the graft SCN were nevertheless able to restore circadian gene expression. Importantly, however, this took much longer to achieve, in contrast to the almost instant response of VIP-null hosts, and these non-VIP signals were unable to drive atypical periods in the host SCN 56. By applying specific blockers to receptors for either arginine vasopressin (AVP) or GRP (neuropeptides secreted by the shell and core SCN, respectively), it was possible to demonstrate that a hierarchy of neuropeptidergic signals underpins this paracrine regulation, with a pre-eminent role for VIP augmented by contributions from AVP and GRP. Moreover, it was possible to show that this interneuronal signalling is sufficiently powerful to maintain circadian pacemaking in the arrhythmic Cry-null SCN, which is deficient in essential elements of the transcriptional negative-feedback loops. Paracrine interneuronal signals can therefore compensate for the genetic deficiency of SCN neurones 56,57. Thus, a hierarchy of paracrine neuropeptidergic signals acts as a bridge between cell- and circuit-level circadian pacemaking in the SCN (Fig. 5).

## Synchronisation of SCN neurones by G-protein coupled signalling and its manipulation by DREADD-mediated pharmacogenetics

How might VIP act on SCN cells and how does this relate to the daily cycles of cAMP, [Ca^2+^]_i_ and CRE activation? VPAC2 is a G-protein coupled receptor that is positively linked to both Gs (to activate cAMP synthesis) and Gq (to stimulate phospholipase C and thereby increase [Ca^2+^]_i_) 58. Consistent with this, the circadian cycle of [Ca^2+^]_i_ is lost in SCN lacking VIP, although individual cells continue to oscillate but without synchrony across the circuit (Fig. 2a). A similar effect is seen for neuronal rhythms of CRE activation: rhythmic but asynchronous, low amplitude circadian oscillations in the absence of VIP (Fig. 2b). It is therefore possible to construct a simple model for neuropeptidergic coupling in the SCN in which VIP is secreted as an output of the TTFL, and in a paracrine fashion then activates a cascade triggered by VPAC2, Gq, PLC, [Ca^2+^]_i_ and finally CRE sequences in *Per* genes of downstream target neurones (Fig. 5). The TTFL of these target cells is therefore phase-locked to that of the VIP neurones. In this model, the differential phasing of cellular gene expression that gives rise to the wave may result from delays in the progression/propagation of the VIP signal, although its origin is more likely more complex. For example, the co-culture studies revealed a contribution of AVP signalling to synchronisation and so the complexity of the wave might arise from reciprocal and sequential signalling from VIP to AVP, and then from AVP to VIP cell populations, in a self-sustaining, perpetual wave.

**Fig. 6:**
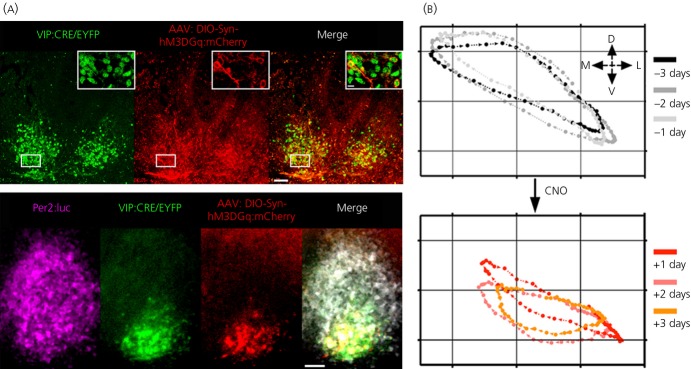
DREADD-mediated activation of Gq signalling in vasoactive intestinal peptide (VIP) neurones re-programmes the circuit-level encoding of circadian time. (a) Upper panel: CRE recombinase-mediated, targetted expression of Gq DREADD to VIP neurones of a suprachiasmatic nucleus organotypic slice. Lower panel: combined bioluminescent and fluorescent imaging of a SCN slice in which Gq-active DREADD was expressed in VIP neurones. Scale bar = 50 μm. (b) X–Y plot of the wave of bioluminescent circadian gene expression rhythms (expressed as the centre of mass of bioluminescence) in an organotypic SCN slice for 3 days before (above) and 3 days after (below) activation of Gq signalling in VIP neurones. Note the change in trajectory to a more ventral location following Gq activation. D, dorsal; L, lateral; M, medial; V, ventral.Redrawn with permission from Maywood *et al*. 56

One way to uncover the mechanisms that couple the SCN neurones into this coherent self-sustaining circuit is to manipulate the respective G-coupled signalling cascades and then monitor the effects in individual cells and across the SCN as a whole. DREADDs comprise a pharmacogenetic tool that offers this utility. They are derived from the muscarinic receptor but modified so that they respond exclusively to a novel ligand, CNO, and activate one of Gs, Gq or Gi. CNO has no endogenous targets in mammalian tissues. This selective activation by CNO can be observed in SCN neurones transduced with lentiviruses encoding DREADD (driven by a neurone-specific promoter) and the CRE-luciferase reporter. Addition of CNO activates CRE in neurones expressing Gs or Gq, and suppresses it in neurones expressing Gi, consistent with known actions of cAMP and [Ca^2+^]_i_ on CRE activity. Importantly, the Gq-mediated activation of CRE also progresses across the SCN circuit into neurones that do not express the DREADD, revealing an indirect trans-synaptic effect whereby Gq activation in some neurones triggers CRE-mediated transcription in their target cells 45. Indeed, at the network level, sustained activation of Gq in a minority of SCN neurones desynchronises cellular rhythms of [Ca^2+^]_i_ and also re-programmes the spatio-temporal wave of *Per* and *Cry* gene expression across the SCN. The re-programmed state is maintained even after Gq activation is terminated by washout of CNO, and is dependent on intrinsic VIP signalling. In VIP-deficient host SCN transduced with DREADD and whose rhythmicity is driven by a nontransduced graft, CRE activation is acutely induced by CNO, confirming the response of Gq in the host SCN. Re-programming of the rhythm, however, does not occur in the absence of intrinsic VIP within the host SCN. VIP therefore mediates the observed Gq-[Ca^2+^]_i_–dependent plasticity of the circuit. To confirm this central role of VIP neurones in this re-programming, intersectional genetics was used, combining SCN slices from a VIP-CRE recombinase mouse line with AAV encoding a flexed Gq DREADD. This ensured that the DREADD was expressed exclusively in VIP neurones (Fig. 6a). Direct activation of Gq signalling in these cells re-programmed the rhythm of *Per* gene expression as effectively as with nontargetted DREADDs: period was lengthened and amplitude was reduced, and the spatio-temporal dynamics of the wave was re-programmed (Fig. 6b). These results therefore demonstrate that a Gq-[Ca^2+^]_i_–VIP axis sits at the heart of circuit-level encoding of circadian time by the SCN. Furthermore, they reveal the potential for intersectional pharmacogenetic approaches to determine the contribution of defined neuronal populations to the ensemble behaviour of the SCN circadian pacemaker.

## Future prospects?

As noted above, the SCN and its circadian functions provide a remarkably clear and robust exemplar for the localisation of function in the central nervous system of mammals. Extending from the behaviour and properties of a few core genes, it is possible to explain circadian phenotypes at all levels of organisation: neurone, transcriptome, neural circuit, whole organism and ecological niche. Further developments in our understanding can be anticipated from the application of approaches similar to those considered in the present review. With growing resolution and certainty, it will be possible to demonstrate how defined neuronal populations control particular behaviours and neuroendocrine states: but what about developments beyond the SCN and the translational value of circadian knowledge? All biology pivots about the efficient use of energy, and the roles of SCN and local clocks in optimising metabolism by temporally integrating energy utilisation across the daily cycle of feeding and fasting is becoming clear. If obesity and diabetes arise not solely as a result of what is eaten, but also when it is consumed (as appears to be the case), then incorporation of the dimension of circadian time will provide important insights into the mechanisms and management of metabolic disease. The feeding/fasting cycle in turn is a product of the cycle of sleep and wakefulness: the most profound change in the neural state and again a product of the SCN. Moreover, it has now been demonstrated that reduced sleep is positively correlated with obesity 59,60. The ultimate pay-off of circadian biology, therefore, will be to unravel the neural mechanisms that effect this regular alternation in our very being and its impact upon physiology. By analogy with circadian integration in the periphery, it can be envisaged that local clocks throughout the brain co-ordinate local rhythms of gene and protein expression, ensuring that neurones produce factors necessary for the contrasting demands imposed on them by wakefulness (online sensory and motor computations) and sleep (recovery, growth and consolidation of information). These local clocks will, in turn, be entrained to each other and to solar time by the SCN. Temporal misalignment of these local clocks will compromise the global state of wakefulness or sleep, causing both to be less efficient. Cognitive defects and mood disturbances will ensue. Although in its infancy, the use of circadian approaches to increase our understanding of neuronal function and states of consciousness offers a direct pathway to identifying ‘how the brain works’.

## Supporting Information

The following supplementary material is available:

**Video S1.** Composite video of GCaMP3-reported [Ca^2+^]_i_ and *Per1-luc* expression (individual channels and merge) in an organotypic suprachiasmatic nucleus slice recorded for 4 days.
